# Oscillation induced propagation of synchrony in structured neural networks

**DOI:** 10.1186/1471-2202-14-S1-P390

**Published:** 2013-07-08

**Authors:** Sven Jahnke, Raoul-Martin Memmesheimer, Marc Timme

**Affiliations:** 1 Network Dynamics, Max Planck Institute for Dynamics & Self-Organization, Göttingen, Germany; 2 Bernstein Center for Computational Neuroscience Göttingen, Göttingen, Germany; 3 Fakultät für Physik, Georg-August-Universität Göttingen, Göttingen, Germany; 4 Donders Institute, Department for Neuroinformatics, Radboud University, Nijmegen, Netherlands

## 

Spatio-temporally coordinated patterns of spiking activity have been experimentally observed in a range of neural circuits, but their dynamical origin is still not well understood. A prominent hypothesis states that propagating synchronized activity through embedded feed-forward networks might dynamically generate such patterns [1,2]. Modeling studies indeed showed that such "synfire-chains" embedded in random recurrent networks enable reliable signal transmission by propagating localized (sub-network) synchrony, if their structure is strongly pronounced compared to the embedding network. This requires in particular a dense connectivity between the neuronal layers of the chain or strongly enhanced synapses and modified response properties of neurons within the chain [3]. So far, however, such prominent large-scale structures have not been experimentally observed.

Single neuron experiments [4] indicate that neuronal dendrites are capable of nonlinearly amplifying sufficiently synchronous inputs by eliciting dendritic spikes, thereby inducing non-additive interactions. Here we demonstrate that such non-additive coupling promotes guided synchrony propagation already in random recurrent neural networks with mildly enhanced, biologically plausible sub-structures and without anatomically superimposed feed-forward chains [5]. Our analysis explains the mechanisms underlying robust propagation and shows in which sense non-additive enhancement -- a local neuron property that dynamically changes with input synchrony -- may complement dense and non-local structural connectivity.

Most neuronal circuits exhibit oscillations of various frequencies and amplitudes [6]. Such oscillations may influence the dynamics of synchrony propagation. We thus further study this influence for both externally induced oscillations as well as for oscillations generated by the rhythmically propagating synchronous activity itself. We find that in networks with linear dendrites and balanced input, the oscillations hinder synchrony propagation, if they effect the dynamics at all. In contrast, for non-additive coupling, oscillations support synchronous propagation, if they are in resonance and the interplay between oscillations and propagating activity induces complex locking patterns: We show that in recurrent circuits containing high-connectivity (hub-)neurons, the oscillatory echo to propagating synchrony can generate synchrony in the remainder of the network and thereby in turn stabilize or even enable synchrony propagation along predefined paths: The network echo promotes signal transmission.
